# In vivo CRISPR/Cas9-mediated screen reveals a critical function of TFDP1 and E2F4 transcription factors in hematopoiesis

**DOI:** 10.1038/s41375-024-02357-w

**Published:** 2024-07-23

**Authors:** Ngoc Tung Tran, Robin Graf, Ernesto Acevedo-Ochoa, Janine Trombke, Timm Weber, Thomas Sommermann, Claudia Salomon, Ralf Kühn, Klaus Rajewsky, Van Trung Chu

**Affiliations:** 1 Max‐Delbrück‐Center for Molecular Medicine in the Helmholtz Association (MDC), Immune Regulation and Cancer, Berlin, Germany; 2 Charité - Universitätsmedizin Berlin, Berlin, 13125 Germany; 3 Max‐Delbrück‐Center for Molecular Medicine in the Helmholtz Association (MDC), Genome Engineering & Disease Models, Berlin, Germany; 4 Present Address: Department of Pediatrics, Herman B Wells Center for Pediatric Research, Indiana University School of Medicine, Indianapolis, IN USA; 5 Present Address: Muscle Research Unit, Experimental and Clinical Research Center, a cooperation between the Max‐Delbrück‐Center for Molecular Medicine in the Helmholtz Association and the Charité ‐ Universitätsmedizin, Berlin, Germany; 6 Present Address: Biobank OWL (BOWL), Medical School OWL, Bielefeld University, Bielefeld, Germany; 7 Present Address: Dynamic42 GmbH, Jena, Germany

**Keywords:** Haematopoietic stem cells, Haematopoiesis

## Abstract

Hematopoiesis is a continuous process of blood cell production driven by hematopoietic stem and progenitor cells (HSPCs) in the bone marrow. Proliferation and differentiation of HSPCs are regulated by complex transcriptional networks. In order to identify transcription factors with key roles in HSPC-mediated hematopoietic reconstitution, we developed an efficient and robust CRISPR/Cas9-based in vivo genetic screen. Using this experimental system, we identified the TFDP1 transcription factor to be essential for HSPC proliferation and post-transplant hematopoiesis. We further discovered that E2F4, an E2F transcription factor, serves as a binding partner of TFDP1 and is required for HSPC proliferation. Deletion of TFDP1 caused downregulation of genes associated with the cell cycle, with around 50% of these genes being identified as direct targets of TFDP1 and E2F4. Thus, our study expands the transcriptional network governing hematopoietic development through an in vivo CRISPR/Cas9-based genetic screen and identifies TFDP1/E2F4 as positive regulators of cell cycle genes in HSPCs.

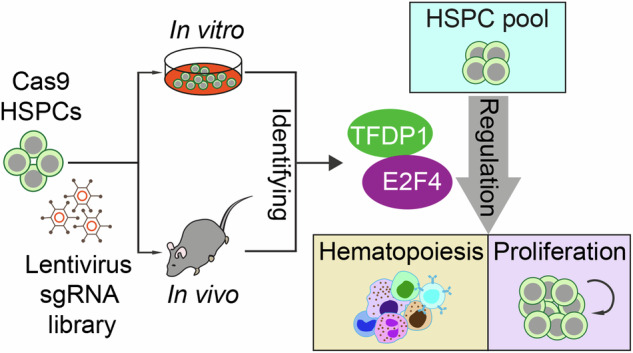

## Introduction

Hematopoietic stem cells (HSCs) are multipotent stem cells that can regenerate all types of blood cells upon transplantation [1]. Native hematopoiesis is driven by continuous differentiation emerging from HSCs and long-lived multipotent progenitors (MPPs). HSCs depend on proliferation and self-renewal in order to maintain the HSC compartment size despite cell loss via differentiation and cell death [2]. Given their important role in day-to-day hematopoiesis, MPPs depend on proliferation and input from HSCs. Thus, both the maintenance of HSC and MPP compartments and the differentiated lineages emerging from these stages are strictly dependent on properly regulated proliferation. In addition to the dependency of HSCs on their normal proliferation rate at steady-state, HSCs must undergo cellular expansion to achieve reconstitution of the entire blood and immune system following transplantation in myeloablated recipients [2].

Several transcription factors of the zinc finger protein (Zfp) superfamily, such as the GATA family [3], Gli1 [4], Gfi1 and Gfi1b [5], Ikaros [6], KLF4 [7], and Sp3 [8], are important in the regulation of cell proliferation and differentiation in hematopoiesis. More recently, the Zfp90 [9], Zfp521 [10], and Zfp145 [11] transcription factors were also found to regulate HSPC proliferation and differentiation. However, this analysis of zinc finger genes may be incomplete, and additional members of this gene family may be involved in the control of these processes.

HSC transplantation into myeloablated hosts has been the mainstay for the in vivo analysis of HSC function. Recent fate mapping experiments shed light on native hematopoiesis [12, 13], but these approaches are less suitable for a rapid analysis of mutations compared to HSC transplantation or in vitro readouts. Along these lines, high-throughput shRNA-mediated knockdown screens in vitro have been performed to identify genes important for self-renewal and maintenance of mouse HSPCs in culture [14]. To screen regulators of HSPC repopulation upon transplantation, an in vivo shRNA-mediated screen has been conducted in mouse HSPCs, and 17 genes affecting HSPC engraftment were identified [15]. However, this technique may suffer from low sensitivity and biases due to the incomplete knockdown of the target genes [16]. Recently, we and others have developed efficient gene mutagenesis in mouse HSPCs and immune cell lineages using a CRISPR/Cas9-mediated approach [17, 18]. This approach leads to rapid and efficient gene deletion and allows genetic analysis of hematopoiesis in vivo upon HSPC transplantation.

Here, we established an in vivo CRISPR/Cas9-based mutagenesis screen that is based on a Cas9 transgenic mouse line, sgRNA-expressing lentiviruses, bone marrow transplantation, and deep sequencing to identify transcription factors with key roles in HSPCs. We validated the hits found in these experiments by testing single sgRNAs in HSPC transplantation experiments. Using this approach, we show that the transcription factors TFDP1 and E2F4 interact in HSPCs and both positively regulate the proliferation of these cells, and hence hematopoiesis.

## Materials and methods

### Mice and ethics approval

Rosa26-Cas9iGFP (Cas9-GFP) mice, C57BL/6, and Rag2^-/-^γc^-/-^ mice were bred in-house and kept in specific pathogen-free facilities. 8–16 weeks-old mice were used. All animal experiments were approved by the Landesamt für Gesundheit und Soziales Berlin (G0110/16, G0140/18).

### Cell culture

HEK293T cells were maintained in DMEM^+/+^ (Gibco) supplied with 10% FCS (Biochrom). HSPCs were cultured in StemSpan^TM^ SFEM II medium (Stemcell) supplied with either a low-cytokine cocktail: mouse SCF (25 ng/ml), mouse TPO (25 ng/ml), and mouse Flt3L (25 ng/ml) (Peprotech) or a high-cytokine cocktail: mouse SCF (50 ng/ml), mouse TPO (50 ng/ml), mouse Flt3L (50 ng/ml), and human IL-11 (50 ng/ml). Primary B cells were co-cultured with 40LB feeder cells as previously described [17].

### sgRNA design and cloning

We used our in-house-developed CrispRGold [17] program to design sgRNAs targeting genes in the library (Table S1). This program allowed us to design specific sgRNAs with high gene editing efficiency. We selected three sgRNAs per gene with high activity for in vitro and in vivo genetic screens. sgRNAs were cloned individually into pKLV-U6_sgRNA-PGK_Puro_2A_mCherry (Addgene #67977) or MSCV-U6_sgRNA-PGK_Puro_T2A_mCherry vectors.

### High-throughput lentivirus production and infection

2 × 10^4^ HEK293T cells were seeded per well of a 96-well plate in 100 μl of complete DMEM^+/+^ medium. One day later, these cells were transfected with the following plasmids: psPAX2 (100 ng), pMD2.G (10 ng), and pKLV-U6-sgRNA-mCherry (100 ng) using Fugene HD (Promega). The next day, the transfected cells were washed once with PBS, and 120 μl of StemSpan^TM^ SFEM II medium was added to each well. 48 h post transfection, 100 μl of virus supernatant were collected and stored at −80 °C.

For lentivirus infection, HSPCs were isolated from the bone marrow of Cas9-GFP and C57BL/6 mice using a FACS sorter (BD), mixed at a 1:1 (in vitro screens) or 3:2 (in vivo screens) ratio, and cultured in the 96-well plate at a density of 2 × 10^4^ cells per well in 100 μl of complete StemSpan^TM^ SFEM II medium supplied with cytokines. The cell mixture was infected with individual sgRNA-expressing lentiviruses in the 96-well plate setup by adding 100 μl of virus supernatant to the well.

### HSPC transplantation

24 h after lentivirus infection with a transduction efficiency of 30%, Cas9^+^/Cas9^-^ HSPCs (3:2 ratio) from different wells were pooled and counted. 5 × 10^4^ transduced HSPCs (3 × 10^4^ of Cas9^+^ and 2 × 10^4^ of Cas9^-^) were mixed with 2 × 10^6^ Rag2^-/-^γc^-/-^-derived BM carrier cells. Cells were washed twice with PBS and resuspended in 200 μl PBS for intravenous injection into a sub-lethally irradiated (5 Gy) Rag2^-/-^γc^-/-^ mouse and 3 recipient mice were used for each screening experiment. The sgRNA coverage in Cas9^+^ and Cas9^-^ HSPCs is calculated as 600 and 400, respectively. Eight weeks after transplantation, BM, splenic, and peritoneal cells from three transplanted recipients per experiment were pooled, and HSPCs, granulocytes, B, T, NK, and CD11b^+^ cells were analyzed and sorted for isolating genomic DNA (Fig. S2B and Table S2).

### Antibodies and FACS analysis

Single-cell suspensions isolated from the bone marrow, spleen, or peritoneal cavity of experimental animals were blocked with TruStain FcX™ antibody for 10 min and stained with specific antibodies (Table S4) for 15 min. The stained cells were washed with FACS buffer (PBS/1% BSA) and analyzed using a BD Fortessa. PI (Propidium Iodide) and DAPI (4’,6-Diamidino-2-Phenylindole) were used to exclude dead cells. The data were analyzed using FlowJo®. For cell sorting, the stained cells were sorted into 15 ml Falcon tubes and centrifuged before DNA and RNA isolation.

### Proliferation and apoptosis assays

Cas9-HSPCs were labeled with 5 μM cell-trace violet (Invitrogen) at 37 °C for 15 min, washed with serum-free StemSpan^TM^ SFEM II medium, and cultured at a density of 2 × 10^5^ cells per 1 ml of StemSpan^TM^ SFEM II medium supplied with the high cytokine cocktail. 6–12 h later, these cells were infected with sgRNA-expressing lentiviruses targeting *Tfdp1*, *E2f4*, or *Rosa26*. Proliferation rates were measured by flow cytometry on days two and four after cell-trace labeling. For the apoptosis assay, the transduced HSPCs were stained with Annexin-V antibody using the Apoptosis Detection Kit (Biolegend, Cat# 640932). To detect active Caspase 3^+^ in apoptotic HSPCs, the transduced HSPCs were selected with 5 μg/ml of Puromycin for three days and then active Caspase 3 was intracellularly stained with Alexa 647 anti-active Caspase 3 antibody (BD biosciences, Cat# 560626) using the BD fixation/Permeabilization kit (BD biosciences, Cat# 554714).

### Colony forming unit cell assay for *Tfdp1* KO

Three days post infection with sgRNA-expressing lentiviruses targeting *Tfdp1* or *Rosa26* gene, mCherry^+^ HSPCs were sorted out and seeded on the MethoCult medium (M3434, Stemcell technologies) following the manufacturer’s protocol. After ten days, the number of colonies was manually counted.

### Western blotting and immunoprecipitation

Cas9-HSPCs were transduced with sgRNA-expressing lentiviruses and selected with Puromycin (5 μg/ml, Sigma) for three days. The transduced HSPCs were then lysed in RIPA buffer (20 mM Tris-HCl (pH 7.5), 150 mM NaCl, 1% NP-40, 0.1% SDS, 0.1% Sodium deoxycholate, and 1 mM EDTA) supplied with protease inhibitors. HSPC lysates were fractionated by SDS-PAGE and transferred to PVDF membranes (GE Healthcare). TFDP1, E2F4, E2F1, and beta-Actin proteins were detected using primary antibodies: mouse anti-TFDP1 (Thermo Scientific, Cat# MA5-11268), mouse anti-E2F4 (Proteintech, Cat# 67812-1-Ig), rabbit anti-E2F4 (Sigma, Cat# AV31175), mouse anti-E2F1 (Proteintech, Cat# 66515-1-Ig) and mouse anti-β-Actin (Sigma-Aldrich Cat# A2228). The primary antibodies were developed with HRP-conjugated goat anti-rabbit and rabbit anti-mouse IgG antibodies (Southern Biotech).

For immunoprecipitation, Cas9-HSPCs were expanded in StemSpan^TM^ SFEM II medium supplied with the high cytokine cocktail for four days. 1 × 10^7^ HSPCs were lysed in 1 ml of RIPA buffer supplied with protease inhibitors. 500 μg of total proteins were immunoprecipitated with primary antibodies: rabbit anti-TFDP1 (Thermo Scientific, Cat# PA5-86135), mouse anti-E2F4 (Proteintech, Cat# 67812-1-Ig), and mouse anti-E2F1 (Proteintech, Cat# 66515-1-Ig) overnight. IP complexes were captured using Protein A + G magnetic beads (Sigma, Cat# 16-663).

### Preparation of the sgRNA library

To perform sgRNA library preparation and deep sequencing, sgRNA barcodes from genomic DNA of the sorted cells were amplified by PCR using KAPA HIFI HotStart Mix (Applied Bio Science) with gene-specific primers including molecular barcodes and Illumina adapter sequences (Table S1), and the following PCR conditions: 95 °C for 3 min; 25 cycles (95 °C for 30 s, 63 °C for 30 s, 72 °C for 30 s) and 72 °C for 5 min. PCR products per sample were pooled and purified using AMPure XP beads (Beckman Coulter) and quantified using a Qubit dsDNA HS assay kit (Invitrogen). For multiplexing sgRNA libraries, these PCR products were indexed through a second PCR with Nextera XT DNA Library Preparation Kit v2 set A (Illumina) using KAPA HIFI HotStart Mix (Applied Bio Science) and the following PCR conditions: 95 °C for 3 min; 10 cycles (95 °C for 30 s, 63 °C for 30 s, 72 °C for 30 s) and 72 °C for 5 min. Indexed PCR products were cleaned using AMPure XP beads (Beckman Coulter), quantified using a Qubit dsDNA HS assay kit (Invitrogen), normalized to 10 ng/μl, and pooled. The amplicon libraries were sequenced using Illumina HiSeq 4000.

### RNA-seq

Total RNA was extracted from sorted HSPCs using the RNeasy Qiagen kit (Qiagen). RNA-seq library preparation and sequencing were done by Novogene (Novogene). Additional methods for RNA-seq analysis are provided in Supplemental Information.

### Computational analysis

Survival/proliferation score was defined as log2 fold change of mCherry^+^GFP^+^/mCherry^-^GFP^+^ cells. sgRNA frequencies were determined using a customized script. The counts for every sgRNA sequence were normalized to the total number of reads per library. The abundance of each sgRNA was defined by the log2 fold change of GFP^+^/GFP^-^ cells. Additional methods for meta-analysis of published available data are provided in Supplemental Information.

### Statistical analysis

At least two independent experiments were performed with several technical replicates for each experiment. The number of biological and technical replicates for individual experiments is outlined in the figure legends or shown as data points in the figures.

## Results

### In vitro CRISPR/Cas9-based screen identifies gene candidates important for HSPC expansion

**Fig. 1:**
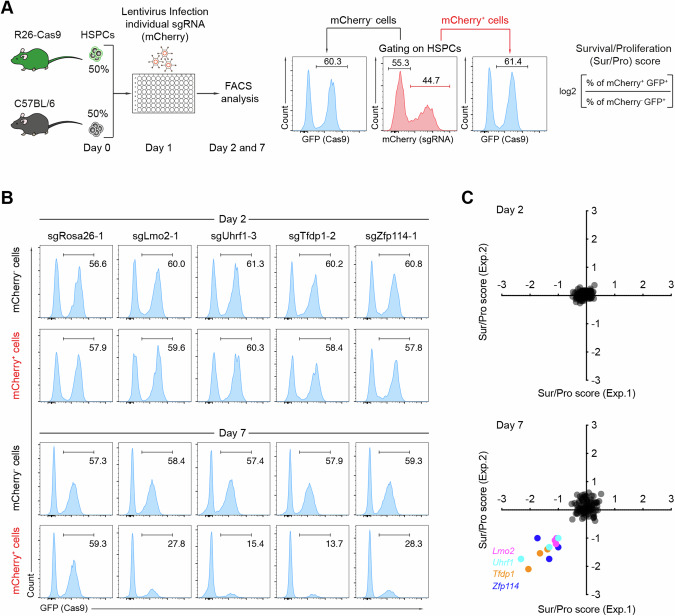
In vitro CRISPR/Cas9-based screen to identify determinants for HSPC expansion. **A** Scheme of CRISPR/Cas9-based screen in mouse HSPCs. Cas9-expressing HSPCs were isolated from R26-Cas9iGFP mice, mixed with C57BL/6 (WT) HSPCs at a ratio of 1:1, and activated for one day. The activated HSPCs were infected with lentiviral particles expressing specific sgRNA in 96-well plates. These cells were cultured and analyzed on day two and seven after infection by flow cytometry. The survival/proliferation (sur/pro) score of the pre-gated HSPCs was defined as indicated. **B** Representative FACS analysis of the frequency of GFP^+^ (Cas9^+^) cells within mCherry^+^ (sgRNA^+^) or mCherry^-^ (sgRNA^-^) HSPCs two (top) and seven days (bottom) post transduction. In each column, the indicated sgRNA was used. **C** Correlation of survival/proliferation scores of two independent experiments at the indicated time points (*n* = 2 biological replicates). Genes with a survival/proliferation score <−1 are highlighted.

The transcriptional control of HSPC proliferation and differentiation, and hence hematopoiesis, remains to be fully explored. Based on publicly available RNA-seq data (Immgen.org), we selected 29 Zfps, 2 members of the TFDP (transcription factor DP) family, and additional transcription factors that are highly expressed in HSPCs for a genetic screen (Fig. S1A and Table S1). To assess the functions of these genes in mouse HSPCs, we first conducted an in vitro CRISPR/Cas9-based screen using three sgRNAs per gene that we designed with CrispRGold [17]. As a negative control, we selected three sgRNAs targeting intron 4 of the *Rosa26* locus. As positive controls, we designed sgRNAs targeting *Lmo2* (LIM domain only 2) [19, 20] and *Uhrf1* (Ubiquitin Like With PHD And Ring Finger Domains 1) [21], two transcription factors known to be essential for HSPC expansion and hematopoiesis. Each sgRNA was cloned into a lentiviral vector expressing mCherry as a reporter. We then isolated Lineage^-^Sca1^+^cKit^+^ HSPCs cells from Cas9-GFP (GFP^+^) and wild-type (WT) C57BL/6 (GFP^-^) mice, mixed them at a ratio of 1:1 and cultured them in 96-well plates. Every well was infected with lentiviruses expressing one sgRNA at a transduction efficiency of 20–60% (Fig. S1B). After two and seven days in culture, we analyzed the frequencies of mCherry^+^GFP^+^ (knockout (KO)) and mCherry^-^GFP^+^ (control) cells and defined a score for survival/proliferation (sur/pro) (Fig. 1A and Fig. S1C). As expected, two days post infection, all sur/pro scores remained comparable for input and control sgRNAs. In contrast, seven days post infection, the sgRNAs against four genes led to a strong reduction in HSPC survival/proliferation (sur/pro score <−1). This gene set included *Lmo2*, *Uhrf1*, *Tfdp1* (transcription factor DP-1), and *Zfp114* (zinc finger protein 114) (Fig. 1B, C and Table S2).

### In vivo screen identifies *Tfdp1* to be indispensable for hematopoiesis

**Fig. 2:**
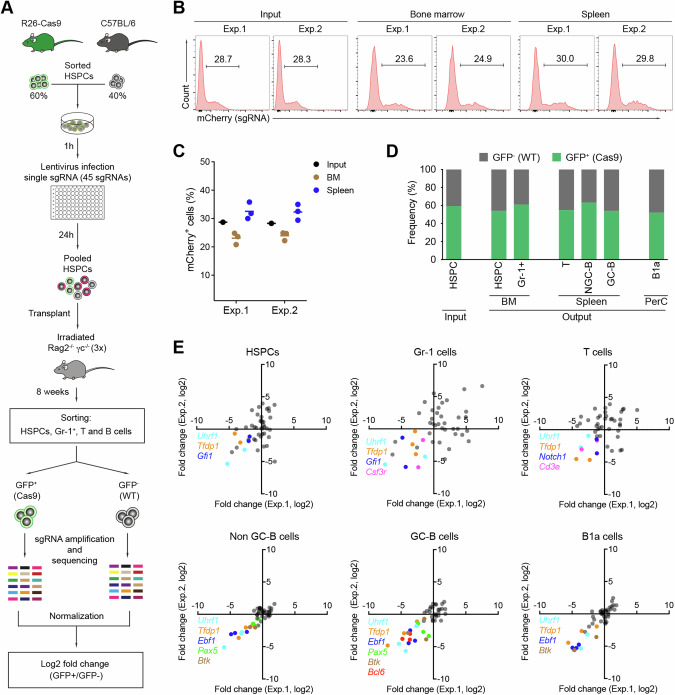
In vivo CRISPR/Cas9-mediated screen to identify regulators of hematopoiesis. **A** Scheme of in vivo CRISPR/Cas9-mediated screen. GFP^+^/GFP^-^ HSPCs were infected with individual sgRNA-expressing lentiviral particles in 96-well plates, pooled, and transplanted to immunodeficient mice (*n* = 3, technical replicates). Eight weeks post transplantation, the sgRNA abundance in HSPC, Gr-1^+^, T, and B cells was analyzed by deep sequencing and used to define a fold change (log2) of GFP^+^ versus GFP^-^. **B** FACS analysis of total mCherry^+^ cells (sgRNA^+^) in the input HSPCs and in cells isolated from the bone marrow and spleens of recipient mice from two independent experiments (*n* = 2, biological replicates). **C** Summary of the data in (**B**). **D** Frequencies of GFP^+^ (Cas9) versus GFP^-^ (WT) cells in input and analyzed immune cell subsets from the bone marrow (BM), spleen, and peritoneal cavity (PerC) of the transplanted animals. **E** Correlation graphs of the sgRNA abundance fold changes (log2) from two independent experiments in the indicated immune subsets. Genes having an impact on the depletion of the respective cell subset are highlighted.

To develop a stringently controlled in vivo CRISPR/Cas9-based screen, we included several genes known to be important for HSPCs (*Gfi1* [22, 23] and *Uhrf1* [21]), B cells and germinal center (GC) B cells (*Ebf1* [24], *Pax5* [25], *Btk* [26], and *Bcl6* [27]), T cells (*Cd3e* [28] and *Notch1* [29]), and granulocytes (*Csf3r* [30]), as well as two negative control genes (*Gfi1b* and *Rosa26*). In addition to the identified positive hits (*Tfdp1* and *Zfp114*), we also included the genes *Mllt3* and *Dach1*, since recent evidence suggests that they may have an impact on hematopoiesis [31, 32]. Hence, the final library consisted of 45 sgRNAs targeting 15 genes (Fig. S2A). We then sorted HSPCs from the bone marrow of Cas9-GFP and C57BL/6 mice, mixed them at a ratio of 3:2, and infected them as in the in vitro screen (Fig. 1A). 24 h after infection, the transduced HSPCs were pooled and transplanted into irradiated immunodeficient Rag2^-/-^γc^-/-^ mice with coverage of ~500 HSPCs per sgRNA (Fig. 2A). To ensure that only one sgRNA is transduced per cell, infection efficiencies were controlled to be lower than 30% [33]. As expected, eight weeks post transplantation, the percentages of total mCherry^+^ cells in the bone marrow and spleen of the recipient mice were in the range of 23–30% and thus comparable to the input infection rates (Fig. 2B, C). Moreover, the ratio of GFP^+^/GFP^-^ cells in various immune cell populations isolated from the bone marrow, spleen, and peritoneal cavity, remained at approximately 3:2, indicating that Cas9^+^ and Cas9^-^ HSPCs equally engraft and repopulate immune cell lineages in the recipient mice (Fig. 2D).

To compare the sgRNA abundance in Cas9^+^ and Cas9^-^ cells in each cell lineage, we sorted the mCherry^+^GFP^+^ and mCherry^+^GFP^-^ population from bone marrow HSPCs, and Gr-1^+^ cells, splenic T, non-GC B and GC-B cells, and peritoneal B1a cells 8 weeks post transplantation (Fig. S2B and Table S2). The frequencies of the virally-inserted sgRNA sequences were then analyzed by deep sequencing. The abundance of each sgRNA was calculated by the log2 fold change of the reads in GFP^+^/GFP^-^ cells (Fig. 2A). As a quality check, we monitored the distribution of individual sgRNAs in the pooled HSPCs before transplantation and found a relatively equal distribution of sgRNAs in the input library (2–9%), suggesting that all sgRNAs were delivered in vivo (Fig. S2C). By analyzing sgRNA deep sequencing for GFP^+^ and GFP^-^ output lineages, we indeed were able to recover all delivered sgRNAs in both sorted GFP^+^ and GFP^-^ fractions at eight weeks post reconstitution. Next, we analyzed the positive control sgRNAs and confirmed that the frequencies of sgRNAs targeting cell lineage-specific genes were strongly reduced in Cas9^+^ cells of the respective cell lineages. This was the case for HSPCs (*Uhrf1* and *Gfi1*), Gr-1^+^ cells (*Uhrf1*, *Gfi1*, and *Csf3r*), T cells (*Notch1* and *Cd3e*), and the B cell lineage (*Ebf1*, *Pax5*, *Btk*, and *Bcl6*), indicating that our in vivo screen is functional and reproducible (Fig. 2E). Consistent with the in vitro screen, disruption of *Tfdp1* led to an impaired development of all analyzed immune cell lineages, indicating crucial roles of this gene in hematopoiesis in vivo (Fig. 2E). In contrast, disruption of *Zfp114*, *Mllt3*, or *Dach1* did not affect the development of any of the analyzed immune cell lineages, indicating that this transcription factor is dispensable for post-transplant hematopoiesis (Table S2). Thus, our in vivo screen provides a robust and reliable system to identify important genes in HSPC expansion and hematopoiesis.

### TFDP1 interacts with E2F4 in mouse HSPCs

In mammals, two TFDP proteins, TFDP1 and TFDP2, have been identified to form heterodimers with E2F family proteins. The E2F-TFDP complexes are crucial for regulating proliferation, differentiation, and apoptosis. The *E2f* family consists of eight genes and contains transcription activators (E2F1-3) [34] and transcription repressors (E2F4-8) [35–40]. Thus, the availability of E2F proteins and their binding partners may contribute to a cell type-specific activation or suppression of cell cycle progression [41, 42].

**Fig. 3:**
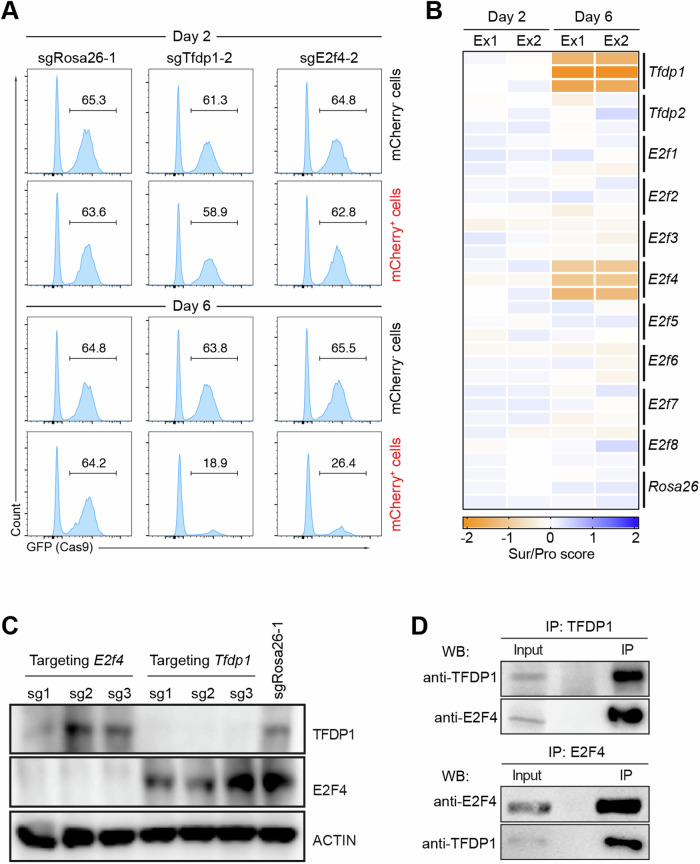
In vitro CRISPR/Cas9-mediated screen to identify E2F members important for HSPC expansion. **A** FACS analysis of the percentage of GFP^+^ (Cas9^+^) cells within the mCherry^+^ (sgRNA^+^) and mCherry^-^ (sgRNA^-^) HSPCs on day two (top) and six (bottom) post transduction. **B** Heatmap of survival/proliferation scores in HSPCs treated with the indicated sgRNAs (*n* = 2, biological replicates). The color indicates decreased (orange) and increased (blue) survival/proliferation scores. **C** Western blot of TFDP1 and E2F4 three days post targeting with sgRNAs against *Tfdp1* and *E2f4*. SgRosa26-1 was used as a negative control. Actin was used as a loading control. **D** Co-immunoprecipitation (Co-IP) using TFDP1 (top) and E2F4 as precipitating antibody (bottom) (*n* = 2, biological replicates).

Our screen revealed that TFDP1 is a crucial transcription factor controlling HSPC proliferation and/or survival. To find functional E2F binding partners of TFDP1 in HSPCs, we next performed an in vitro CRISPR/Cas9-based screen targeting all members of the *E2f* family in mouse HSPCs. Consistent with our previous screens, *Tfdp1* was essential for HSPC survival/proliferation. Strikingly, the deletion of *E2f4* had a similar outcome as the deletion of *Tfdp1*, whereas the sgRNAs against other *E2f* members showed no effect (Fig. 3A, B). All sgRNAs targeting *Tfdp1* and *E2f4* led to efficient gene KO (~90%) in HSPCs three days post transduction (Fig. 3C). To address whether the effects of TFDP1 and E2F4 on HSPCs are cell type-specific, we performed the CRISPR/Cas9-mediated KO of these genes in activated B cells. *Tfdp1* KO caused a strong reduction of B cell numbers, while the ablation of *E2f4* only led to a modest reduction, suggesting that the importance of *E2f4* is cell type-specific (Fig. S3A, B). In HSPCs, *Tfdp1* and *E2f4* are the most highly expressed E2F family members in mouse HSPCs based on RNA-seq (Fig. S3C). To assess whether E2F4 directly interacts with TFDP1 in HSPCs, we performed co-immunoprecipitation (co-IP) assays and observed that the two proteins indeed co-immunoprecipitate (Fig. 3D). In addition, E2F1, a transcription activator that regulates cell cycle progression in numerous cell types [43, 44], also directly interacts with TFDP1 in HSPCs (Fig. S3D). However, HSPC survival/proliferation was not affected by the knockout of this gene (Fig. 3B). Based on the RNA-seq data and Western blotting, the loss of TFDP1 did not alter the expression levels of E2F4 in KO HSPCs and vice versa (Fig. S3C and S3E). Thus, E2F4 directly interacts with TFDP1 in HSPCs and both TFDP1 and E2F4 are critical for HSPC proliferation and/or survival in vitro.

### *Tfdp1* and *E2f4* are critical for post-transplant hematopoiesis

**Fig. 4:**
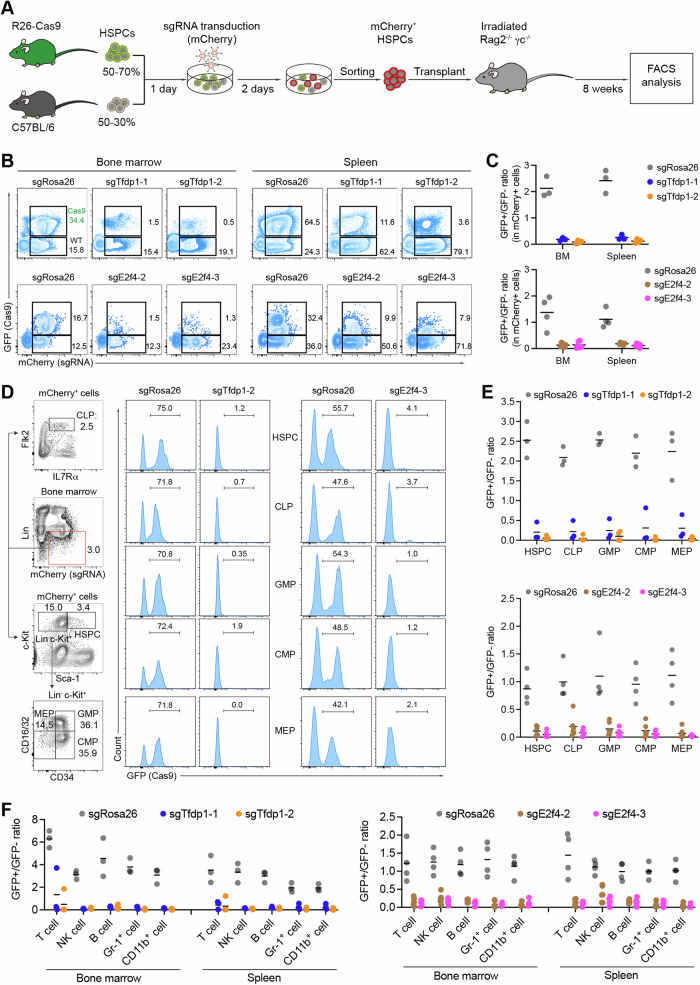
Functional validation of *Tfdp1* and *E2f4* in hematopoiesis in vivo. **A** Scheme of in vivo functional validation in hematopoiesis. **B** Representative FACS analysis of mCherry^+^GFP^+^ (KO) and mCherry^+^GFP^-^ (WT) donor cells in the bone marrow (BM) and spleen of the recipients transplanted with the indicated sgRNA-treated HSPCs. **C** Summary of the GFP^+^/GFP^-^ ratios within mCherry^+^ cells in the bone marrow (BM) and spleen of recipient animals as shown in (**B**) (*n* = 3–6, technical replicates). **D** FACS gating strategy (left) and GFP analysis (right) in the indicated stem and progenitor cell populations: HSPC, common lymphoid progenitor (CLP), granulocyte/myeloid progenitor (GMP), common myeloid progenitor (CMP), megakaryocyte/erythroid progenitor (MEP). The used sgRNAs are indicated at the top. **E** Summary of the GFP^+^/GFP^-^ ratios calculated in (**D**) from different mice transplanted with sgTfdp1-infected (top) and sgE2f4-infected (bottom) HSPCs. SgRosa26 was used as a control in all experiments. **F** GFP^+^/GFP^-^ ratios in the analyzed mature immune cell lineages: T cells, natural killer (NK) cells, B cells, Gr-1 (granulocyte), and myeloid cells (CD11b^+^) isolated from bone marrow and spleen of mice transplanted with the indicated sgRNA-infected HSPCs.

To validate the function of the *E2f4* and *Tfdp1* genes in hematopoiesis in vivo, we transduced GFP^+^/GFP^-^ HSPCs with lentiviruses expressing sgRNAs targeting *Rosa26*, *Tfdp1*, or *E2f4* gene. Three days after infection, the transduced HSPCs were sorted and transplanted into irradiated Rag2^-/-^γc^-/-^ mice (Fig. 4A). Eight weeks post transplantation, we observed a strong depletion of the (GFP^+^mCherry^+^) *Tfdp1* and *E2f4* KO cells in the bone marrow and spleen of the recipient mice (Fig. 4B, C).

To determine which stem and progenitor cell compartments are affected by the KO of *Tfdp1* and *E2f4*, we analyzed the GFP percentages in the HSPC, CLP (common lymphoid progenitor), GMP (granulocyte/myeloid progenitor), CMP (common myeloid progenitor), and MEP (megakaryocyte/erythroid progenitor) compartments. Strikingly, disruption of *Tfdp1* or *E2f4* led to a strong reduction of KO cells in all analyzed compartments (Fig. 4D, E), causing a depletion of KO cells in the respective cell lineages in the bone marrow and spleen (Fig. 4F, Fig. S4 and S5). Thus, TFDP1 and E2F4 are critical in regulating HSPC proliferation and/or survival in vivo.

### *Tfdp1* and *E2f4* are critical for the proliferation of HSPCs

**Fig. 5:**
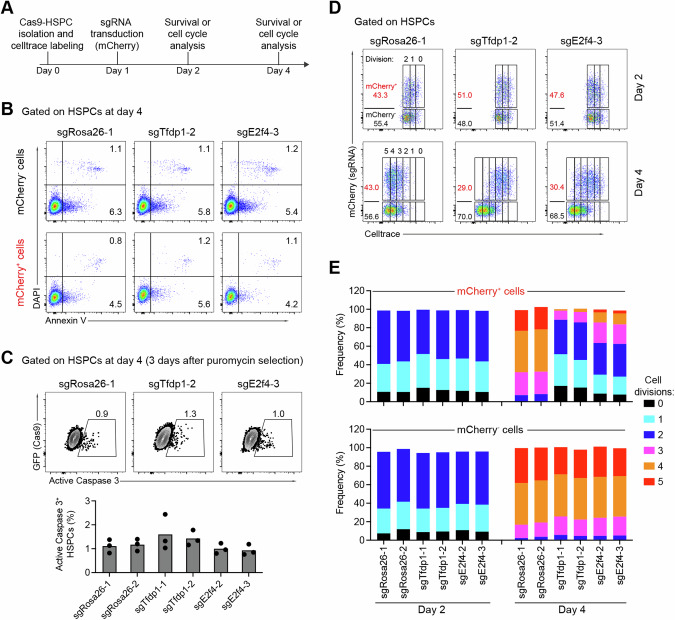
TFDP1 and E2F4 regulate HSPC proliferation, but not apoptosis. **A** Scheme of assessment of HSPC proliferation and apoptosis. Cas9-HSPCs were labeled with Celltrace, cultured for one day and transduced with lentiviral particles expressing sgRNAs targeting *Tfdp1*, *E2f4*, or the *Rosa26*. **B** Representative FACS analysis of the percentages of Annexin V^+^DAPI^-^ (early) and Annexin V^+^DAPI^+^ (late) apoptotic cells within mCherry^-^ (sgRNA^-^, upper panel) or mCherry^+^ (sgRNA^+^, lower panel) HSPCs treated with the indicated sgRNAs. **C** Representative FACS analysis of active Caspase 3^+^ apoptotic HSPCs treated with the indicated sgRNAs three days post puromycin selection (top) and summary of the data (bottom) based on HSPCs from three mice (*n* = 3). **D** Representative FACS analysis of the proliferation rates of mCherry^-^ (sgRNA-) and mCherry^+^ (sgRNA^+^) HSPCs infected with the indicated sgRNAs two and four days post cell-trace labeling. The number of cell divisions is indicated. **E** Percentage of cell division in mCherry^+^ (upper) and mCherry^-^ (below) HSPC subpopulations treated with the indicated sgRNAs on day two and day four post cell-trace labeling (*n* = 3 independent experiments).

To address whether the E2F4 and TFDP1 transcription factors regulate the proliferation or apoptosis in HSPCs, we performed cell-trace labeling and Annexin-V staining assays. We labeled Cas9-HSPCs with cell-trace violet before infection. The labeled cells were then infected with sgRNA-expressing lentiviruses targeting *Tfdp1*, *E2f4*, or *Rosa26*, and analyzed one and three days post infection (Fig. 5A). No difference was detected between sgRNA-containing and control cells with respect to early (Annexin-V^+^DAPI^-^) and late (Annexin-V^+^DAPI^+^) apoptotic/necrotic cells in all experimental groups (Fig. 5B and Fig. S6A) three days post infection. Furthermore, no significant changes in the frequencies of active Caspase 3^+^ apoptotic HSPCs between KO and control groups were detected three days post puromycin selection (Fig. 5C). Thus, the E2F4 and TFDP1 transcription factors have no detectable impact on apoptosis in the HSPC compartment in vitro.

Next, we assessed the proliferation rates in HSPCs by analyzing cell divisions. One day post infection, there was no difference in the proliferation rates of *Tfdp1*-KO, *E2f4*-KO, and *Rosa26*-KO HSPCs. In contrast, three days post infection, the mCherry^+^ cells containing sgRNAs against *Tfdp1* and *E2f4* showed a significant reduction in proliferation compared to control cells. While the majority of *Rosa26*-targeted HSPCs reached division 5, a significant proportion of *Tfdp1*-KO or *E2f4*-KO HSPCs was arrested at divisions 1 and 2 (Fig. 5D, E and Fig. S6B). Notably, no differences in proliferation rates were observed among the three experimental settings in the non-transduced mCherry^-^ cells (Fig. 5D, E). Consistent with their impaired proliferation, *Tfdp1*-KO HSPCs exhibited a severe reduction in colony-forming cell ability in vitro, both in terms of the number and size of colonies (Fig. S6C).

### Global transcriptional changes in *Tfdp1*-KO HSPCs

**Fig. 6:**
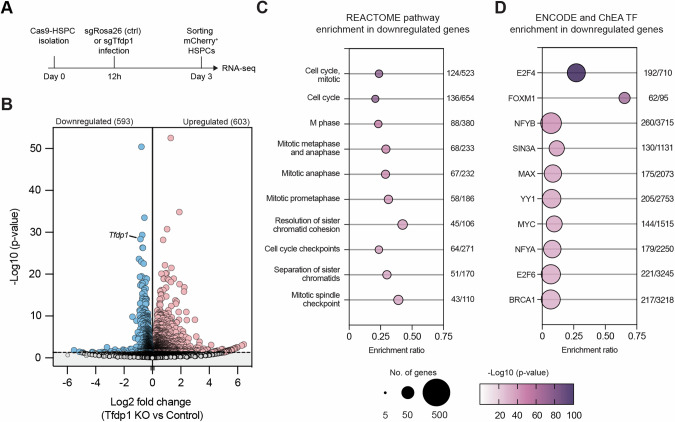
Transcriptional regulation of TFDP1 in HSPCs. **A** Experimental scheme of the RNA-seq experiment in *Tfdp1*-KO HSPCs. As a negative control, sgRosa26 was used. **B** Volcano plot depicting changes in gene expression in *Tfdp1*-KO HSPCs; y-axis represents the log_10_ transformation of the adjusted *p* value and x-axis the log_2_ transformation of the fold change. Red and blue dots represent up and downregulated genes, respectively. REACTOME pathway (**C**) and transcription factor enrichment analysis (**D**) of the downregulated genes in *Tfdp1*-KO HSPCs. The bubble plots depict the top 10 most significantly enriched gene sets. The bubble size corresponds to the number of genes and the color intensity reflects the adj. *p* value for each geneset. The total number of genes found within each dataset and the number of genes present in the downregulated genes are shown on the right.

**Fig. 7:**
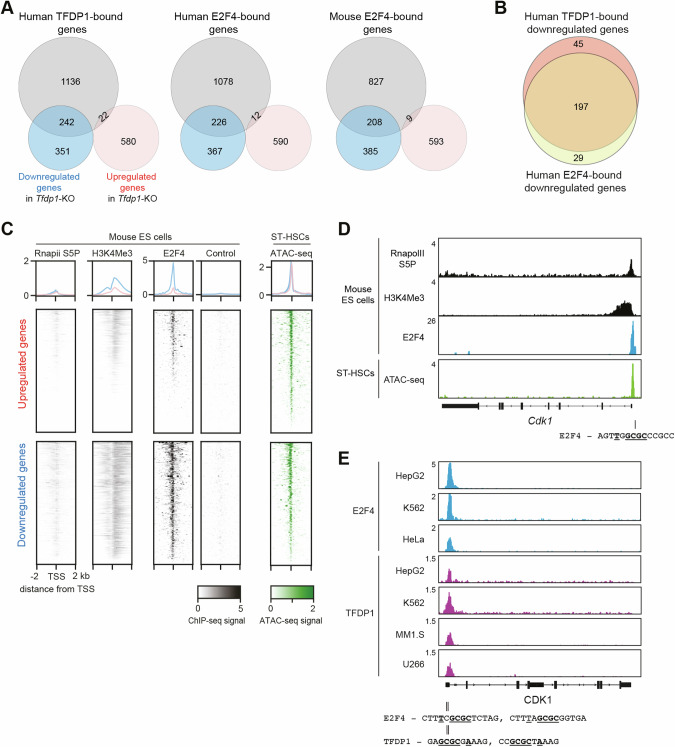
Meta-analysis of the role of TFDP1 and E2F4 in gene activation in mouse HSPCs. **A** Venn diagrams depicting the overlap between the differentially expressed genes in *Tfdp1*-KO HSPCs (downregulated genes in blue and upregulated genes in red) and human TFDP1- (left; GSE80661; GSE105217; GSE127368), human E2F4- (middle; GSE31477; GSE170651), and mouse E2F4- (right; GSE48666) bound target genes. **B** Intersection between human TFDP1- and E2F4-bound genes downregulated in *Tfdp1*-KO HSPCs. **C** Density plots (upper panel) and heatmaps (lower panel) depicting the average tag densities around TSSs (−2/+2 kb) of up- and downregulated genes in *Tfdp1* KO HSPCs. Data are derived from the ChIP-seq of RNA polymerase II S5P (RnapolII S5P; GSE34518), H3K4Me3 (GSE75426), and E2F4 (GSE48666) (together with a negative control) in mouse ES cells. Right panel: ATAC-seq signals (GSE100738) from mouse short-term (ST) HSCs in the same genomic regions. **D** Example of RnapolII S5P, H3K4Me3, E2F4 tracks in mouse ES cells and ATAC-seq in ST-HSCs at the mouse *Cdk1* locus. **E** Example of E2F4 and TFDP1 ChIP-seq signals at the *CDK1* locus in various human cell types. Mouse and human E2F4 and TFDP1 DNA binding sites derived from the Unibind database are shown and the core nucleotides involved in DNA binding are highlighted.

To gain insights into the transcriptional regulation of *Tfdp1* in HSPCs, we performed RNA-seq in sorted *Tfdp1*-KO HSPCs and *Rosa26*-targeted control HSPCs three days post infection (Figs. 6A and 7A). We performed differential gene expression analysis and found 603 and 593 differentially expressed genes (DEGs) to be significantly upregulated and downregulated in *Tfdp1*-KO HSPCs, respectively (Fig. 6B and Table S3). Next, we performed pathway enrichment analysis using the REACTOME database to link changes in gene expression to specific biological pathways. Consistent with the proliferation defect previously observed in *Tfdp1*-KO HSPCs, the downregulated genes showed a significant enrichment for pathways associated with cell cycle and mitosis, whereas the upregulated genes were enriched for biological processes related to immune activation (Fig. 6C and Fig. S7B). Strikingly, the promoters of the downregulated genes were also significantly enriched for E2F4 and FOXM1 binding sites (Fig. 6D). FOXM1 is a known regulator of cell cycle genes [45]. In contrast, the upregulated genes were enriched for binding sites of PU.1 (Spi1), a repressor of E2F signaling [46] (Fig. S7D). These findings suggest that TFDP1 primarily acts as a transcriptional activator in mouse HSPCs, likely through its interaction with E2F4, and regulates gene programs involved in the cell cycle and mitosis.

### TFDP1 and E2F4 control cell cycle genes in mouse HSPCs

To investigate whether TFDP1 and its binding partner E2F4 act as transcriptional activators, we compared our gene expression data with published RNA-seq and ChIP-seq datasets in different mouse and human cell types. For this, we extracted the lists of DEGs in *Tfdp1*-KO mouse HSPCs, *E2f4*-KO mouse embryonic stem cells (ES cells; GSE109684) [47], and *TFDP1*-KO human HAP cells (myeloid leukemia cell line; GSE144453) [48]. In addition, we used the ChIPatlas database to define the sets of top target genes (90th percentile) directly bound by human TFDP1, human E2F4, and mouse E2F4 in various cell types (see Table S3 for references and data sources). Consistent with the previous results and in all analyzed sets, the downregulated genes of the three RNA-seq datasets showed a significant overlap with the TFDP1- and E2F4-bound target genes, whereas the overlap with the upregulated genes was not significant (Fig. 7A and Fig. S8A). The genes that are downregulated and direct E2F4 targets showed a strong overlap with the downregulated genes being TFDP1 targets (>80%), suggesting that TFDP1 and E2F4 act as an activator complex in mouse cells (Fig. 7B). Of note, the promoters of the downregulated genes (385 genes) that did not overlap with the mouse E2F4 target set (Fig. 7A, right), were significantly enriched for Myc binding sites (Fig. S8B), indicating that the impaired proliferation of *E2f4*-KO HSPCs was partially mediated by Myc.

To link these gene set observations in HSPCs to experimental mouse ChIP measurements, we intersected our DEGs with the E2F4 ChIP-seq data from mouse ES cells. Mouse ES cells are transcriptionally similar to HSPCs, and ablation of E2F4 in these cells has caused a similar defect in proliferation [49]. We mapped RNApolII S5P (RNA polymerase II mark of active transcription; GSE34518) [50], H3K4Me3 (histone mark for active transcription start sites (TSSs); GSE75426) [51], and E2F4 (GSE48666) [52] ChIP-seq signals to the TSS of up- and downregulated genes in *Tfdp1*-KO HSPCs (Table S3). Consistent with the above results, we observed a higher E2F4 ChIP-seq signal at the TSSs of downregulated genes compared to the upregulated genes, indicating that these regions may indeed be bound by E2F4 (Fig. 7C). Mapping of ATAC-seq data (GSE100738) [53] from different mouse HSPC populations to the same genomic regions confirmed that these chromatin regions are open in HSPCs (Fig. 7C and Fig. S8C). Among the downregulated genes bound by both TFDP1 and E2F4 (Fig. 7B, right), we found conserved E2F4- and TFDP1-binding motifs that are conserved in mice and humans (Fig. 7D, E). Collectively, our meta-analysis suggests that TFDP1/E2F4 heterodimers directly bind to conserved DNA binding sites and activate the expression of cell cycle-associated genes, thereby regulating HSPC proliferation.

## Discussion

Upon transplantation of HSPCs into myeloablated recipients, donor HSPCs colonize the host bone marrow and subsequently reconstitute the entire blood and immune compartments. While it is challenging to genetically analyze the functions of endogenous HSPCs by targeted mutagenesis beyond individual mutations, HSPCs can be subjected to genetic screens in the context of bone marrow reconstitution before adoptive transfer. We chose this approach to introduce selected sgRNAs together with Cas9 into primary HSPCs. Importantly, the screen was set up to include wild-type control HSPCs that were analyzed side-by-side with the mutated HSPCs in downstream in vitro and in vivo analysis. Using this approach, we identified TFDP1 and E2F4 to play an essential role in HSPC proliferation, and consequently hematopoietic reconstitution. Although homeostatic and post-transplant hematopoiesis are not identical, this result strongly indicates that TFDP1 and E2F4 are critical also for hematopoietic development in situ, by interfering with HSPC proliferation.

E2Fs 1 to 6 bind to DNA as heterodimers in association with their dimerization partners, TFDP1 and TFDP2, whereas E2F7 and E2F8 do not require TFDPs to bind to DNA [54]. The classical E2Fs (E2F1, E2F2, E2F3a, E2F3b, E2F4, and E2F5) can physically associate with RB family proteins (RB, p130, and p107) at the transactivation domain. Binding to RB proteins inhibits the transcriptional activity of these E2Fs. The latter are further subdivided into canonical activators (E2F1, E2F2, and E2F3) and repressors (E2F4 and E2F5) based on their association with RB family proteins and their nuclear localization during the G1/S transition of the cell cycle [55]. Although E2F4 is categorized as a canonical repressor of cell cycle progression, recent evidence suggests that it has pro-proliferative effects in certain tissues and cell types. For example, E2F4^-/-^ mice are smaller than wild-type mice and have defects in multiple organs, including blood, skin, and intestinal tissue [56–58]. In addition, E2F4 has been shown to promote the proliferation of fetal liver erythroid cells, embryonic stem cells, and intestinal epithelial cells by directly activating the expression of cell cycle genes [47, 57, 59].

Our experiments demonstrate that TFDP1 and E2F4 directly interact and are both essential for HSPC proliferation. Although E2F1 also interacts with TFDP1 in HSPCs, its deletion did not affect HSPC proliferation, indicating that TFDP1 and E2F4 are activators of the cell cycle in this particular cell type. Accordingly, our gene expression analysis shows that the deletion of TFDP1 leads to a decrease in the expression of cell cycle genes, further supporting the idea that TFDP1/E2F4 heterodimers promote mouse HSPCs proliferation through the regulation of target genes. Indeed, our meta-analysis of publicly available data confirms that this proliferative effect likely occurs through the direct binding and activation of genes associated with the cell cycle and mitosis. Notably, the deletion of E2F4 in mouse ES cells led to a similar proliferation defect that could not be rescued by other E2F members, such as E2F1, underlining the unique role of E2F4 in both HSPCs and ES cells [55].

Although the DNA binding motifs of TFDP1 and E2F4 are highly conserved between mouse and human [60], we show that TFDP1, but not E2F4, is required for the proliferation of activated B cells, suggesting that TFDP1 interacts with other E2F factors in these cells. Thus, the functional composition of the TFDP/E2F complex depends on the cellular contexts. Along these lines, multiple studies have reported that E2F1 is overexpressed in various types of human cancer and exerts pro-proliferative effects likely through the binding and activation of cell cycle genes [61, 62], further indicating the participation of distinct E2F members and other factors regulating the activity of the TFDP1/E2F heterodimers in distinct cellular contexts.

The genetic screen presented in this work is based on CRISPR/Cas9-mediated mutagenesis of mouse HSPCs, their transfer into irradiated immunodeficient Rag2^-/-^γc^-/-^ mice, and the subsequent analysis of their progeny in the hematopoietic system. The limited numbers of cells transferred in such experiments (typically 50 to 100 thousand cells per recipient) set limits to the complexity of the sgRNA library to be screened. In the experiments by Lara-Astiaso et al. [63] and our work, less than 100 sgRNAs were screened, with full and robust recovery in all hematopoietic lineages analyzed post-reconstitution in Cas9 negative controls. LaFleur et al. performed a similar screen with a pooled library of 20,000 sgRNAs, 500 of which were recovered per lethally irradiated C57BL/6 congenic recipient, with very low sgRNA abundance in some cases [64]. Thus, this approach allows to screen thousands of genes by increasing the number of recipient animals. More recently, Haney et al. conducted a screen with 70,000 sgRNAs targeting ~7000 genes by transplanting the mutagenized HSPCs into 17 lethally irradiated C57BL/6 congenic recipients and observed 50% sgRNA recovery in analyzed lineages [65]. While these high-throughput screens are attractive to identify novel genes whose knockouts lead to positive selection, smaller screens can be designed to robustly detect both positive and negative selection with higher sensitivity.

In summary, our study establishes the crucial role of TFDP1 and its interaction with E2F4 in regulating HSPC proliferation and maintaining proper hematopoiesis. Further functional analyses and exploration of specific target genes and downstream signaling pathways regulated by TFDP1 and E2F4 will be required to fully understand the impact of these transcription factors on hematopoiesis and immune function.

### Supplementary information

Supplemental Information

Table S1

Table S2

Table S3

Table S4

## Data Availability

The datasets generated during the current study are available from the corresponding authors upon reasonable request.
