# Supplementary material for: In vivo CRISPR/Cas9-mediated screen reveals a critical function of TFDP1 and E2F4 transcription factors in hematopoiesis
Source: Leukemia. 2024 Jul 23;38(9):2003–15. doi: 10.1038/s41375-024-02357-w (PMC11347378; doi:10.1038/s41375-024-02357-w)
Supplement: Supplementary file 1 — Supplemental Information [file 41375_2024_2357_MOESM1_ESM.pdf]

Supplemental Information

In vivo CRISPR/Cas9-mediated screen reveals a critical function of TFDP1 and E2F4 transcription factors in hematopoiesis

Ngoc Tung Tran, Robin Graf, Ernesto Acevedo-Ochoa, Janine Trombke, Timm Weber, Thomas Sommermann, Claudia Salomon, Ralf Kühn, Klaus Rajewsky and Van Trung Chu

Contents:

Supplemental Materials and Methods;
Supplemental Figure 1;
Supplemental Figure 2;
Supplemental Figure 3;
Supplemental Figure 4;
Supplemental Figure 5;
Supplemental Figure 6;
Supplemental Figure 7;
Supplemental Figure 8

Supplemental Materials and Methods

RNA-seq data analysis

For RNA-seq data analysis, high-quality reads were mapped to the mouse genome (mm10) and differential expression analysis was performed using STAR and DESeq2. The changes in gene expression were considered significant when the adjusted p-value < 0.05 and a Log_2 fold change > 0.37. The FPKM value was used for abundance estimation and comparisons. The over-representation analysis method was implemented with the Enrichr[1] tool to determine pathways and transcription factors (TFs) at the promoter regions of the DEGs using unranked lists of genes based on the previous cut-off applied. The pathway annotations used for the enrichment analysis were taken from REACTOME[2]. The annotations of TFs used were from the "ENCODE and ChEA Consensus TFs from ChIP-X[3]. Only the top 10 gene sets based on their corrected p-values were registered. The heatmaps, volcano, and bubble plots were generated using Prism v.10.2.3.

Meta-analysis of published available data

We collected the transcriptomic data from the GEO database and defined lists of differentially expressed genes based on a Log2 fold change > 0.5 (Table S3). Additionally, we applied the "Target genes" function of the ChIP atlas database[4] to obtain lists of target genes of human and mouse TFDP1 and E2F4 transcription factors. The mean MACS2 p-value (binding score) derived from multiple ChIP-seq experiments was used to define sets of top-bound target genes by selecting the genes on the 90th percentile of the overall distribution. When necessary, mouse gene IDs were converted to human IDs using the biomaRt v3.19[5] tool in R. We performed overlap analysis with all the datasets, including ours, and determined their significance using one-tailed Fisher's exact tests calculated with the "GeneOverlap" package in R[6]. To visualize the

similarity of the lists, we used the Jaccard coefficients calculated in the same package. The Venn diagrams with area-proportional intersections were calculated using the BioVenn web tool and further modified for proper visualization[7].

We used the bedtools2 package[8] implemented through the Galaxy web platform, on the public server (https://www.usegalaxy.org) to analyze public datasets[9]. To map the sequencing data from a given factor, we used the genomic coordinates of the transcription start sites (TSSs) +/- 2kb of up- and downregulated genes from our RNA seq experiment. Next, we obtained the standard-processed Bigwig files and the corresponding peak-calling (q-value <1e-5) of relevant mouse and human TFDP1 and E2F4 ChIP-seq datasets (Chip-atlas.org) or ATAC-seq from different mouse HSPC populations (immgen.org) (Table S3). The heatmaps and summary plots were created using the computematrix function using 50 bp non-overlapping bins for score averaging, and the data was visualized using the plotHeatmap and plotProfile functions. Additionally, we used the Unibind database to obtain the mappings from the binding motifs of TFDP1 and E2F4 that were available for the cell lines selected in this study[10]. Human and mouse ChIP-seq, ATAC-seq, and binding motifs were visualized in the tracks of individual genes using the Integrated Genome Viewer v2.16.2[11]. The genomic features used in this study were based on the mouse reference genome assembly mm10, GRCm38 (https://www.ncbi.nlm.nih.gov/assembly/GCF_000001635.20/) and, human reference genome assembly hg38, GRCh38 (https://www.ncbi.nlm.nih.gov/datasets/genome/GCF_000001405.26/). The gene annotations were based on the NCBI Refseq release 219.

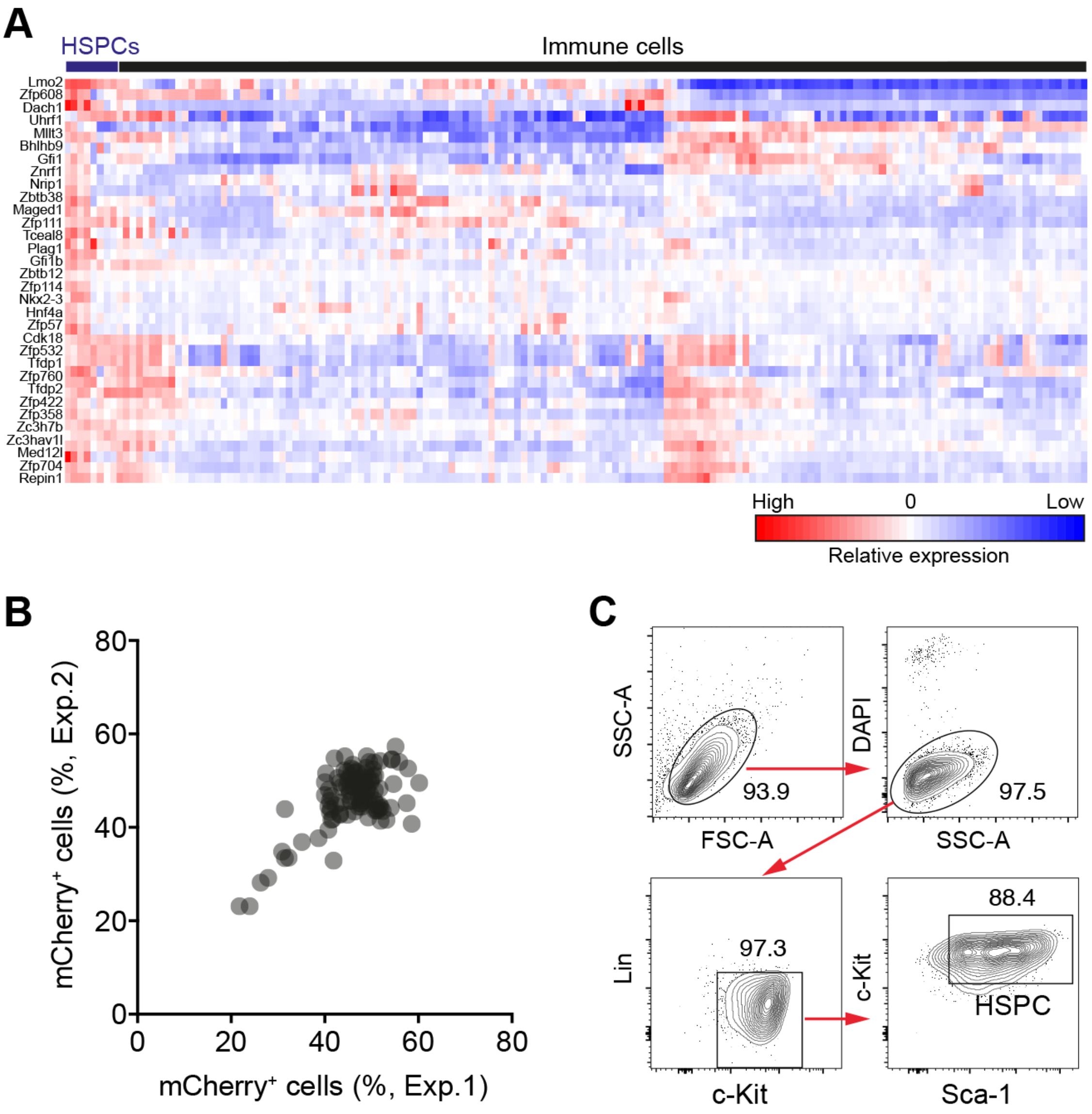

Figure S1. *In vitro* CRISPR/Cas9-mediated screen in mouse HSPCs. (**A**) Relative expression levels of the screened transcription factors in HSPCs and other immune cell lineages. Data were derived from the Immgen database (Immgen.org). (**B**) Frequencies of sgRNA-transduced HSPCs (mCherry$^+$) two days post infection in two independent experiments. Each dot represents an individual sgRNA. (**C**) FACS gating strategy to analyze the HSPC population.

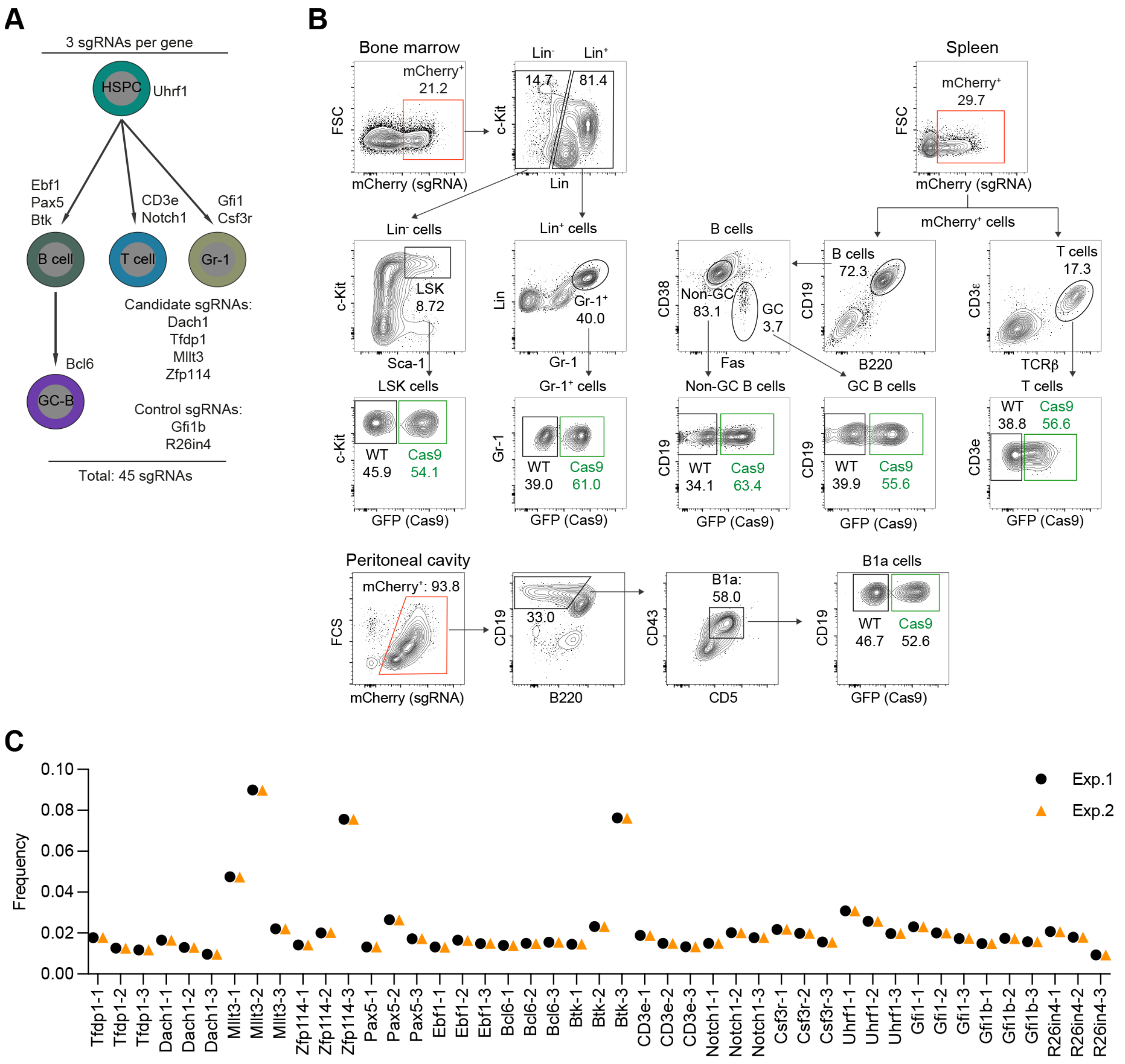

Figure S2. *In vivo* CRISPR/Cas9-mediated screen in mouse HSPCs. (A) Experimental scheme showing the target genes and the number of sgRNAs used in the screening library. The targeted lineage-specific genes that are known to be important for the respective cell population (HSPCs, B, germinal center (GC)-B, T, and Gr-1^{+} cells) are indicated next to the cell population. **(B)** FACS gating strategy showing the ratio of GFP^{+} versus GFP^{-} cells in individual immune cell lineages isolated from the bone marrow (BM), spleen, or peritoneal cavity (PerC) of the recipient animals at 8 weeks after transplantation. **(C)** Deep sequencing analysis of the frequency of each sgRNA in the transduced HSPC pool (input) from two independent experiments.

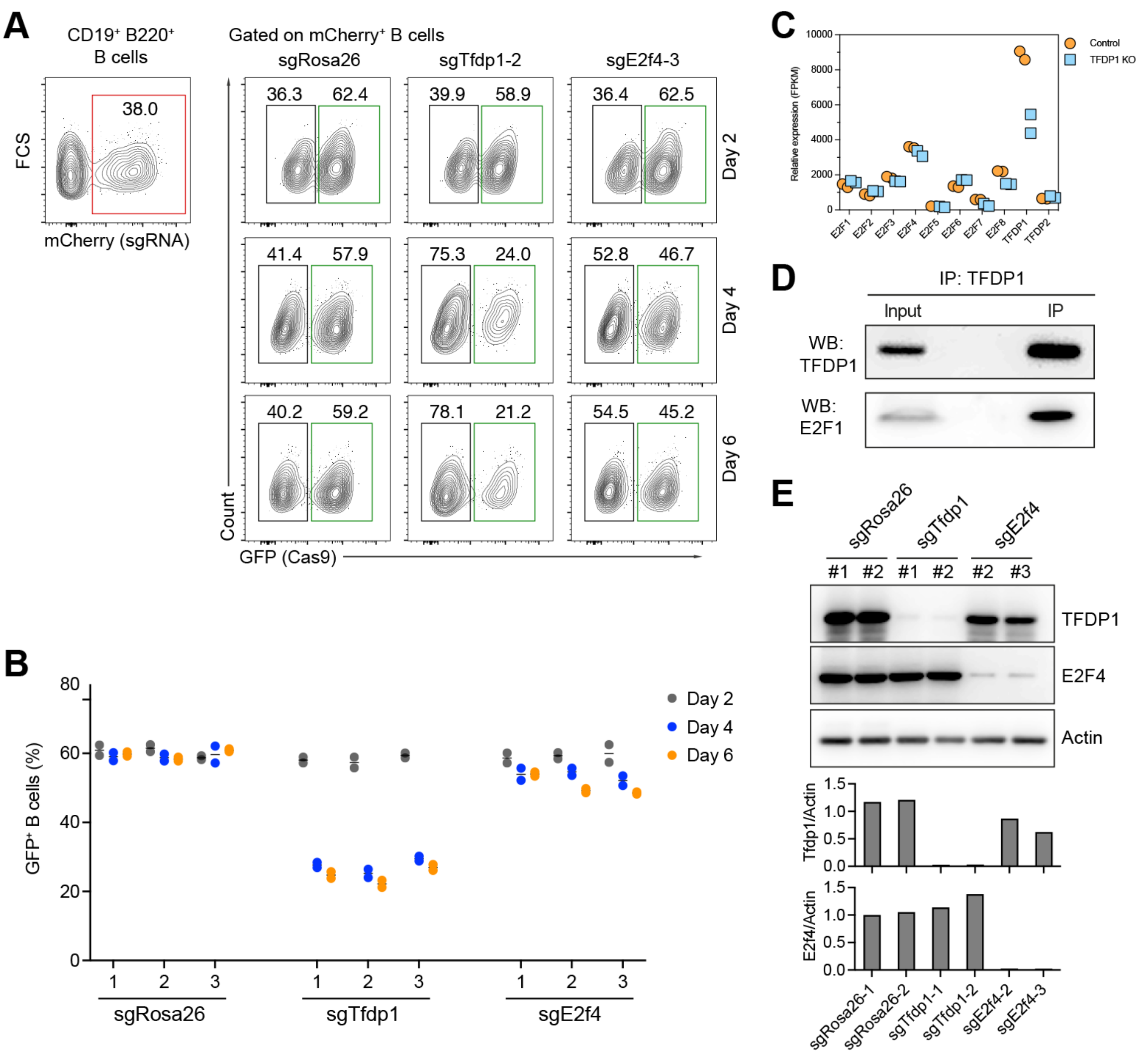

Figure S3. ***In vitro*** **CRISPR/Cas9-mediated knockout of** ***Tfdp1*** **and** ***E2f4*** **in mouse B cells and HSPCs. (A)** Representative pre-gating on the mCherry⁺ population (sgRNA+) (left) and FACS analysis showing the frequencies of GFP⁺ (Cas9) and GFP⁻ (WT) mCherry+ B cells at the indicated time points post targeting *Rosa26*, *Tfdp1*, or *E2f4* gene (right). **(B)** Summary of the frequencies of GFP⁺ B cells depicted in **(A)** from two independent experiments. **(C)** RNA-seq data showing the relative expression of E2F family members in *Rosa26*-targeted (control) and *Tfdp1* (KO) sorted mouse HSPCs. (**D**) Western blot of co-immunoprecipitation of TFDP1 and E2F1 proteins in HSPCs. (**E**) Western blot of TFDP1 and E2F4 in HSPCs treated with the indicated sgRNAs 3 days post puromycin selection. The bar charts show the Actin-normalized signals of TFDP1 (upper panel) and E2F4 (lower panel).

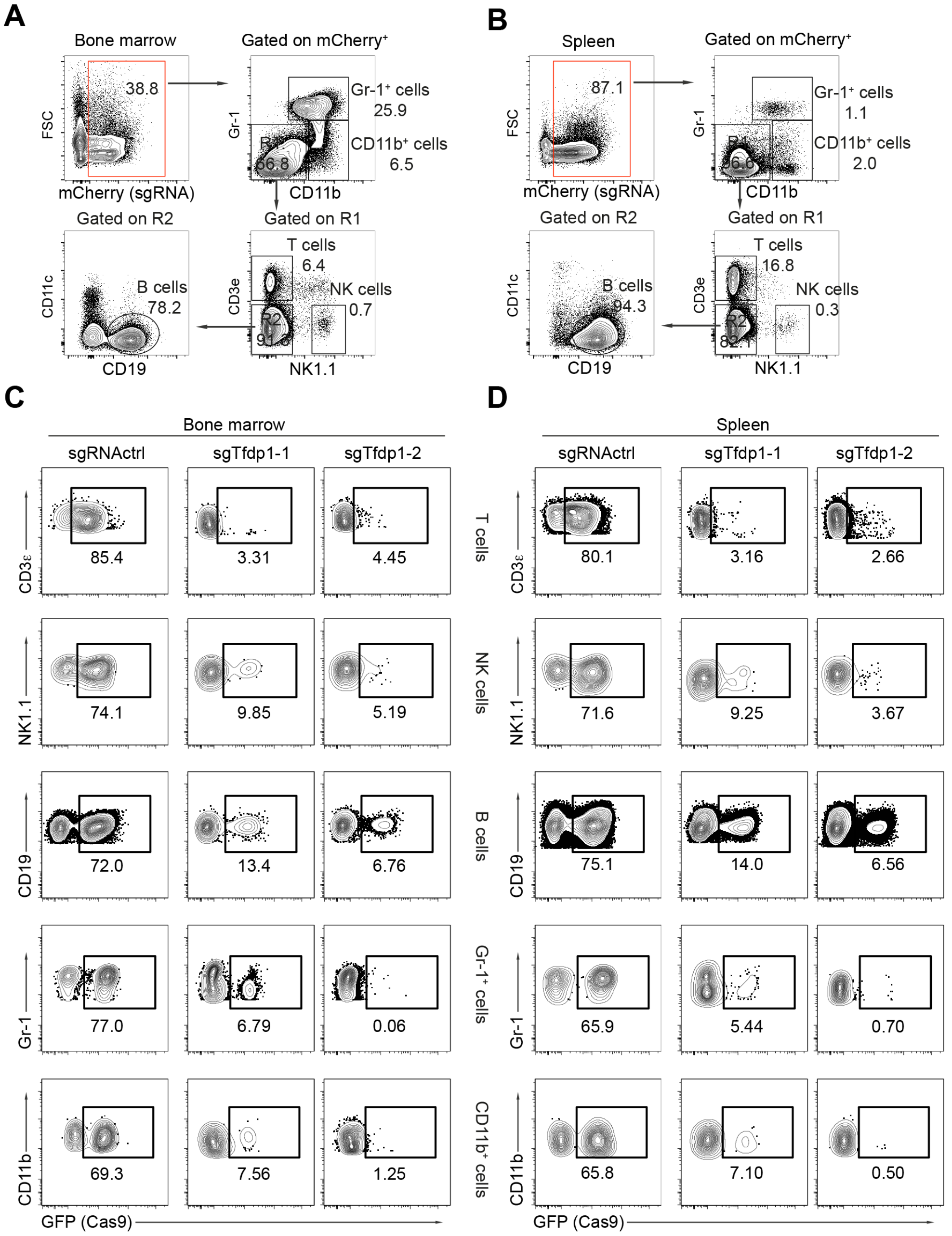

Figure S4. Deletion of *Tfdp1* leads to impaired development of immune cell lineages. FACS gating strategy to identify the indicated mature immune cell subsets in the bone marrow (**A**) and spleen (**B**) of the recipient animals 8 weeks post transplantation. After gating on mCherry^{+} cells, the percentage of Cas9^{+} and Cas9^{-} cells were analyzed in granulocytes (Gr-1^{+}), myeloid cells (CD11b^{+}), T cells (CD3e^{+}), NK cells (NK1.1^{+}), and B cells (CD19^{+}) in the bone marrow (**C**) and spleen (**D**) after treatment with the indicated sgRNAs.

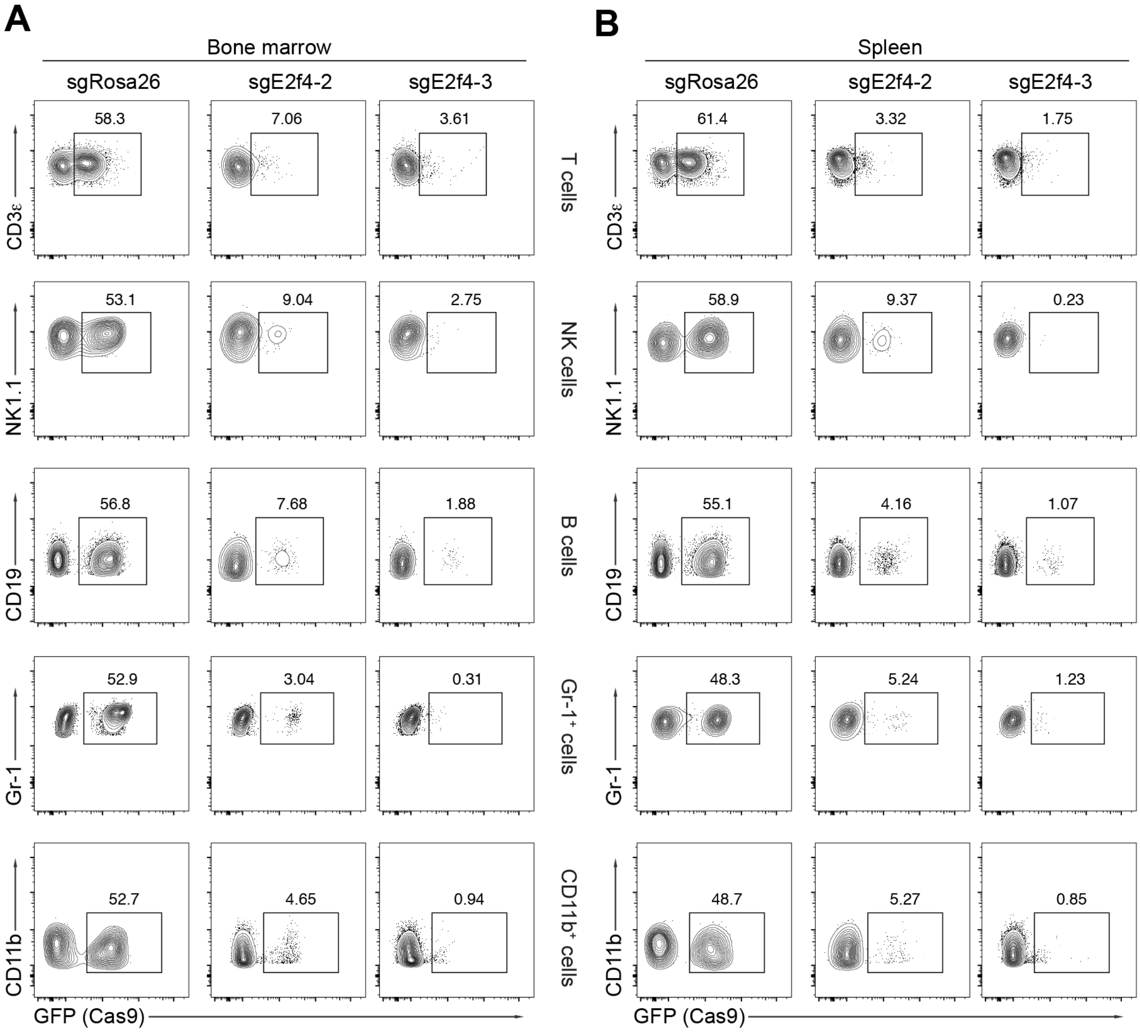

Figure S5. Deletion of *E2f4* leads to impaired development of immune cell lineages. FACS plots of Cas9 (GFP^{+}) cells in T cells (CD3e^{+}), NK cells (NK1.1^{+}), B cells (CD19^{+}), granulocytes (Gr-1^{+}), and myeloid cells (CD11b^{+}) in the bone marrow (**A**) and spleen (**B**) of the recipient mice there were transplanted 8 weeks before with the HSPCs targeted with the indicated sgRNAs.

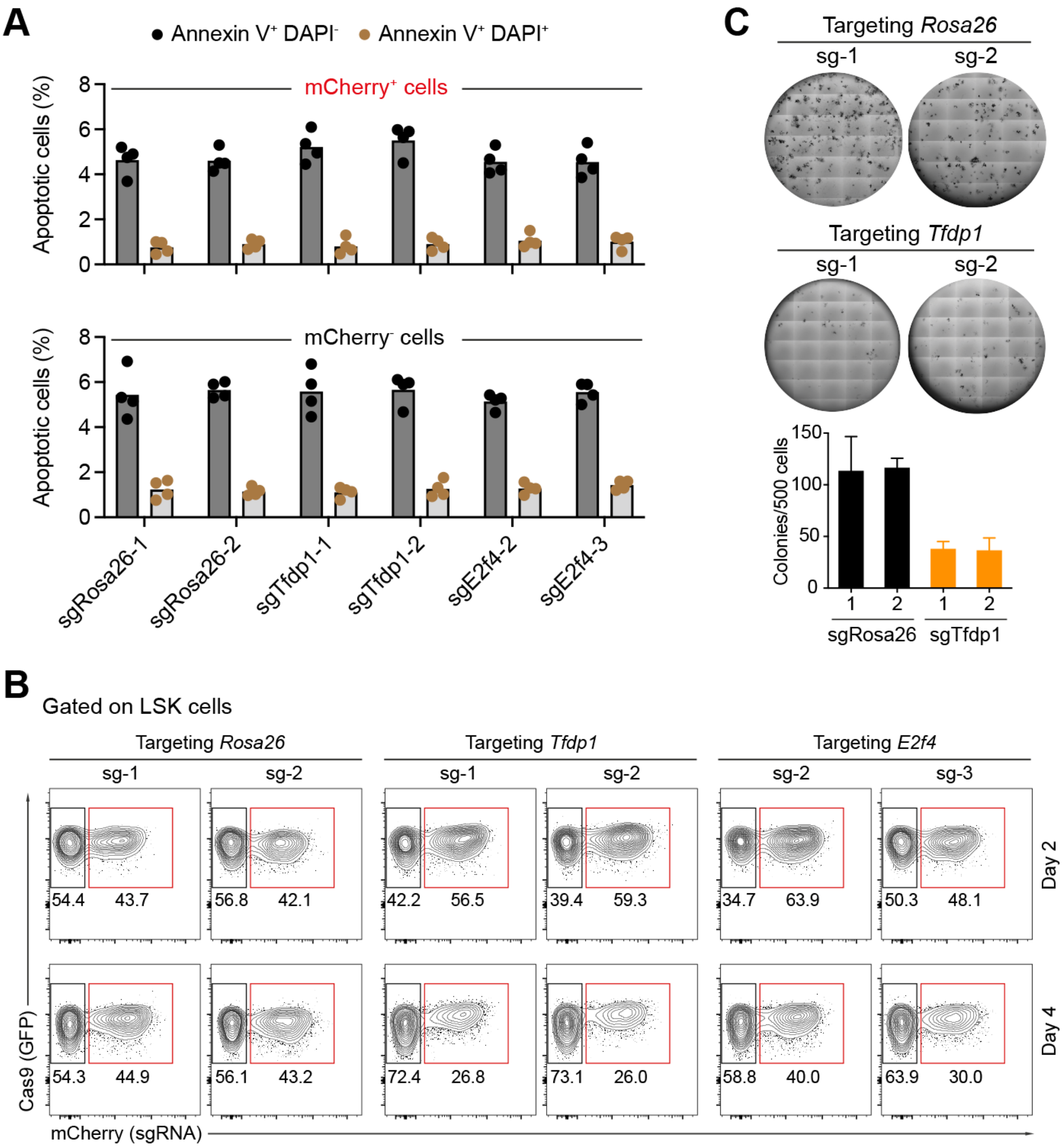

Figure S6. E2F4 and TFDP1 transcription factors are required for HSPC expansion *in vitro*. (A) FACS-based percentages of Annexin V$^+$DAPI$^-$ and Annexin V$^+$DAPI$^+$ apoptotic cells within mCherry$^+$ (sgRNA, upper panel) or mCherry$^-$ (no sgRNA, lower panel) cells treated with the indicated sgRNAs. **(B)** Pre-gated on HSPCs (LSK cells), FACS analysis showing the percentages of mCherry$^+$ cells transduced with sgRNAs targeting *Rosa26* (control), *Tfdp1*, or *E2f4* gene at day 2 and day 4 after transduction. **(C)** Colony forming unit cell assays showing the number of colonies generated by HSPCs transduced with the indicated sgRNAs. The lower panel summarizes the data from three independent experiments.

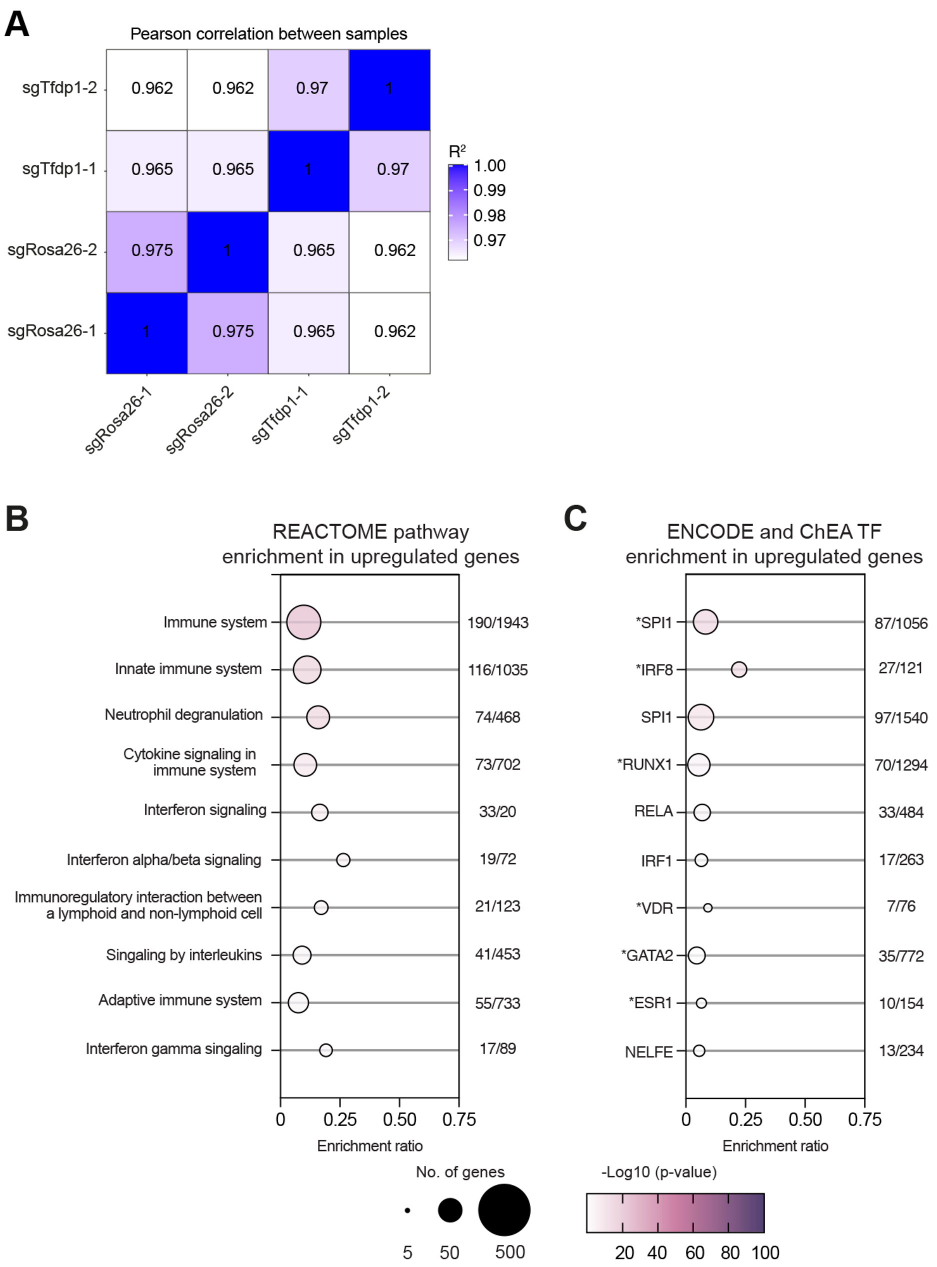

Figure S7. RNA-seq analysis of upregulated genes in *Tfdp1*-KO HSPCs. (A) Correlogram showing the Pearson correlation coefficients between *Tfdp1* KO and control samples (sgRosa26). (**B**) REACTOME pathway enrichment analysis of the upregulated genes after *Tfdp1* KO. (**C**) Transcription factor enrichment analysis of the upregulated genes after *Tfdp1* KO. Both bubble plots show the top 10 gene sets that were significantly affected. The size of the bubbles corresponds to the number of genes, and the color indicates the adj. p-value for each geneset. The total number of genes in each gene set and the number of genes present in the upregulated genes are shown on the right.

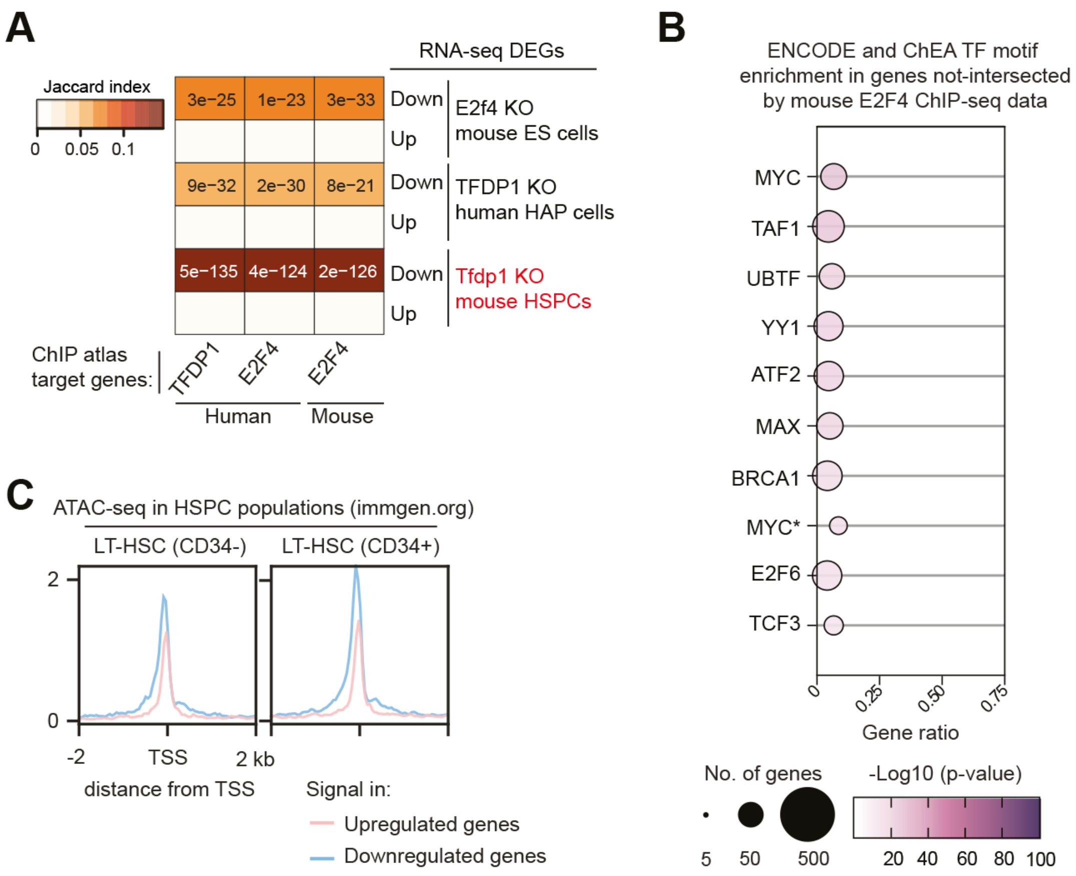

Figure S8. Integrative analysis of differentially expressed genes in *Tfdp1* KO mouse HSPCs using public datasets. (**A**) Heatmap showing the overlap between the gene lists of DEGs (down and up) derived from our (highlighted in red) and public RNA-seq (y-axis) datasets (mouse ES cells: GSE109684, human HAP cells: GSE144453), and the top predicted (90th percentile) TFDP1- (GSE80661; GSE105217; GSE127368) and E2F4- (GSE31477; GSE170651) bound target genes obtained from the ChIPatlas database (x-axis) (see Table S3 for references and data sources). The color scale indicates the Jaccard coefficient as a measurement of the similarity between both lists (0, no similarity; 1, equal). The *p*-values of the Fisher's exact test for each comparison are indicated in each cell. (**B**) Transcription factor enrichment analysis in the downregulated genes after *Tfdp1* KO, that did not overlap with the mouse E2F4 target set (385 non-intersected genes from Fig. 7A). (**C**) Average density plot of the tag distributions across open-chromatin regions derived from ATAC-seq data (GSE100738; immgen.org) obtained from two mouse long-term (LT)-HSC subsets (CD34- and CD34+), around the TSSs +/-2kb of upregulated (red line) and downregulated (blue line) genes after *Tfdp1* KO.

References

1. Kuleshov MV, Jones MR, Rouillard AD, Fernandez NF, Duan Q, Wang Z*, et al.* Enrichr: a comprehensive gene set enrichment analysis web server 2016 update. *Nucleic Acids Res* 2016 Jul 8; **44**(W1)**:** W90-97.

2. Gillespie M, Jassal B, Stephan R, Milacic M, Rothfels K, Senff-Ribeiro A*, et al.* The reactome pathway knowledgebase 2022. *Nucleic Acids Res* 2022 Jan 7; **50**(D1)**:** D687-D692.

3. Lachmann A, Xu H, Krishnan J, Berger SI, Mazloom AR, Ma'ayan A. ChEA: transcription factor regulation inferred from integrating genome-wide ChIP-X experiments. *Bioinformatics* 2010 Oct 1; **26**(19)**:** 2438-2444.

4. Zou Z, Ohta T, Miura F, Oki S. ChIP-Atlas 2021 update: a data-mining suite for exploring epigenomic landscapes by fully integrating ChIP-seq, ATAC-seq and Bisulfite-seq data. *Nucleic Acids Res* 2022 Jul 5; **50**(W1)**:** W175-W182.

5. Smedley D, Haider S, Ballester B, Holland R, London D, Thorisson G*, et al.* BioMart--biological queries made easy. *BMC Genomics* 2009 Jan 14; **10:** 22.

6. Shen L. Test and visualize gene overlaps. *Biocondutor* 2024.

7. Hulsen T, de Vlieg J, Alkema W. BioVenn - a web application for the comparison and visualization of biological lists using area-proportional Venn diagrams. *BMC Genomics* 2008 Oct 16; **9:** 488.

8. Ramirez F, Ryan DP, Gruning B, Bhardwaj V, Kilpert F, Richter AS*, et al.* deepTools2: a next generation web server for deep-sequencing data analysis. *Nucleic Acids Res* 2016 Jul 8; **44**(W1)**:** W160-165.

9. Afgan E, Baker D, van den Beek M, Blankenberg D, Bouvier D, Cech M*, et al.* The Galaxy platform for accessible, reproducible and collaborative biomedical analyses: 2016 update. *Nucleic Acids Res* 2016 Jul 8; **44**(W1)**:** W3-W10.

10. Puig RR, Boddie P, Khan A, Castro-Mondragon JA, Mathelier A. UniBind: maps of high-confidence direct TF-DNA interactions across nine species. *BMC Genomics* 2021 Jun 26; **22**(1)**:** 482.

11. Thorvaldsdottir H, Robinson JT, Mesirov JP. Integrative Genomics Viewer (IGV): high-performance genomics data visualization and exploration. *Brief Bioinform* 2013 Mar; **14**(2)**:** 178-192.
